# Wafer-Based Evaluation of the Effects of Center Frequency and F-Number on Lateral Resolution in Scanning Acoustic Microscopy

**DOI:** 10.3390/s26134058

**Published:** 2026-06-26

**Authors:** Minseok Son, Jincheol Kim, Yuon Song, Juho Kim, Jongmyoung Choi, Jeesu Kim

**Affiliations:** 1 Department of Cogno-Mechatronics Engineering, Pusan National University, Busan 46241, Republic of Korea; sms4493@pusan.ac.kr (M.S.); yuonsong@pusan.ac.kr (Y.S.); 2 Mechatronics Inspection Technology Co., Ltd., Incheon 21999, Republic of Korea; jckim@mit21.co.kr (J.K.); jhkim@mit21.co.kr (J.K.); jmchoi@mit21.co.kr (J.C.)

**Keywords:** scanning acoustic microscopy (SAM), non-destructive testing, ultrasound transducer, lateral resolution

## Abstract

Scanning acoustic microscopy is a useful non-destructive imaging technique for semiconductor inspection, providing acoustic contrast without physical sectioning. However, the selection of an ultrasound transducer for high-quality imaging is not determined by the operating center frequency alone. The focusing condition, represented by the F-number, also plays a critical role in determining the lateral resolution. In this study, the combined effects of the center frequency and F-number on lateral resolution were investigated using wafer-based test samples. Focused ultrasound transducers with different center frequencies were used to image a striped resolution target for quantitative lateral resolution analysis. In addition, a custom-fabricated silicon wafer containing void-mimicking patterns was also imaged for qualitative evaluation. The results show that a higher frequency does not necessarily guarantee better lateral resolution. In fact, a lower-frequency transducer with tighter focusing showed greater image quality compared to a higher-frequency transducer with a larger F-number. These findings indicate that both frequency and F-number should be jointly considered when selecting ultrasound transducers for semiconductor inspection. This wafer-based evaluation provides practical guidance for optimizing imaging conditions in scanning acoustic microscopy, according to target feature size and inspection requirements.

## 1. Introduction

Recent advances in semiconductor manufacturing have led to increasingly dense integration and continued miniaturization of device features [1,2]. This trend requires inspection techniques capable of detecting internal defects such as delamination, voids, micro-cracks, and bonding failures. These defects can critically affect device reliability and manufacturing yield. Therefore, accurate non-destructive detection has become essential for process optimization and quality control in modern semiconductor packaging and wafer manufacturing [3,4].

Among non-destructive testing techniques, scanning acoustic microscopy (SAM) has attracted considerable attention because it can visualize subsurface structures using high-frequency focused ultrasound waves [5,6,7,8]. SAM detects differences in acoustic impedance within a specimen and enables imaging of internal features [9]. Compared with X-ray or scanning electron microscopy, which are conventional imaging techniques for inspection, SAM is non-ionizing, fully non-destructive, and capable of inspecting structures beneath opaque encapsulants, making it particularly attractive for wafer-level and packaged-die inspection [10,11,12]. In addition, SAM can provide depth-resolved information with relatively high spatial resolution, which is advantageous for evaluating semiconductor defects and internal microstructures [13,14,15].

In SAM, spatial resolution is one of the key factors determining image quality. In general, increasing the ultrasound frequency reduces the acoustic wavelength and can therefore improve diffraction-limited lateral resolution [16,17]. However, higher frequencies also increase acoustic attenuation, which may reduce signal penetration and limit the detectable imaging depth. Although the acoustic wavelength in the coupling medium and signal processing methods can affect the effective spatial resolution, the characteristics of the ultrasound transducer, particularly the operating center frequency and focusing geometry, are dominant factors [18,19]. The focusing geometry is commonly characterized by the acoustic F-number ($N_{F}$), defined as the ratio of focal length ($l_{f}$) to aperture diameter (D):

(1) $$N_{F}=\frac{l_{f}}{D}.$$

The lateral resolution ($\delta_{L}$) is conventionally approximated as follows:

(2) $$\delta_{L}=1.02\times N_{F}\times \lambda ,$$

where λ is the wavelength of the acoustic wave. This relationship indicates that the F-number should be considered together with the operating frequency when estimating lateral resolution. In practice, a high-frequency transducer does not necessarily guarantee superior resolution if its F-number is relatively large, whereas a lower-frequency transducer with tighter focusing may provide comparable or even better lateral resolution.

Although this relationship is theoretically well established, practical transducer selection in SAM-based semiconductor inspection is often guided primarily by the operating frequency [20,21]. Therefore, experimental comparisons using wafer-based resolution targets can help clarify how different combinations of center frequency and F-number affect actual imaging performance under comparable measurement conditions. To the best of our knowledge, such wafer-based empirical comparisons remain limited. Therefore, even a representative comparison using commercially available or practically relevant transducers can provide useful reference data for target-specific transducer selection.

In this study, the combined effects of center frequency and F-number on the lateral resolution were experimentally investigated using a custom-designed SAM system. A striped resolution target and a semiconductor wafer containing void-mimicking patterns were imaged using four focused ultrasound transducers with different center frequencies and F-number combinations. The acquired RF signals were input into a consistent signal processing pipeline to generate ultrasound images, enabling a fair comparison of the imaging performance among the transducers. Quantitative lateral resolution was evaluated using the striped resolution target, while qualitative feature visualization was assessed using the wafer specimen. The novelty of this study lies in the wafer-based empirical evaluation of the combined effect of center frequency and F-number under consistent SAM imaging conditions. This empirical comparison provides practical reference data on how operating frequency and F-number jointly influence SAM image resolution to support preliminary transducer selection for target-specific non-destructive semiconductor inspection. The result can also be used as a basis for more systematic evaluations in future studies.

## 2. Materials and Methods

### 2.1. System Configuration

**Figure 1:**
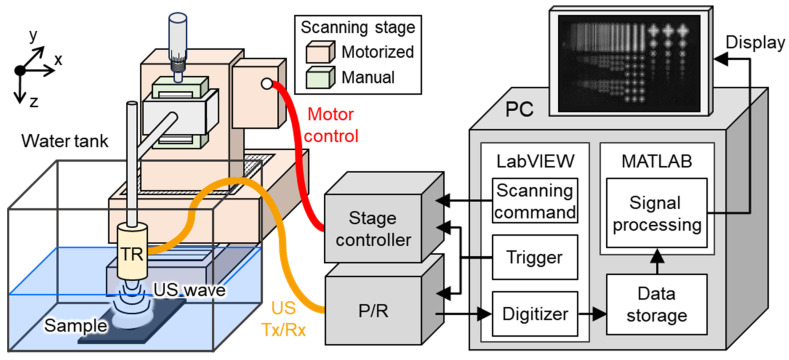
Schematic illustration of the custom-designed SAM system. Among the three linear scanning stages, two axes (with red tint) are motorized for automated raster scanning, while one axis (with green tint) is manually adjusted for sample positioning and focal alignment. The PC controls the scanning sequence, trigger generation, data acquisition, signal processing, and image display. During scanning, pulse-echo ultrasound signals are acquired from the sample and processed in real time to generate SAM images. SAM, scanning acoustic microscopy; US, ultrasound; TR, ultrasound transducer; Tx, transmission; Rx, reception; P/R, pulser/receiver; PC, personal computer.

**Figure 2:**
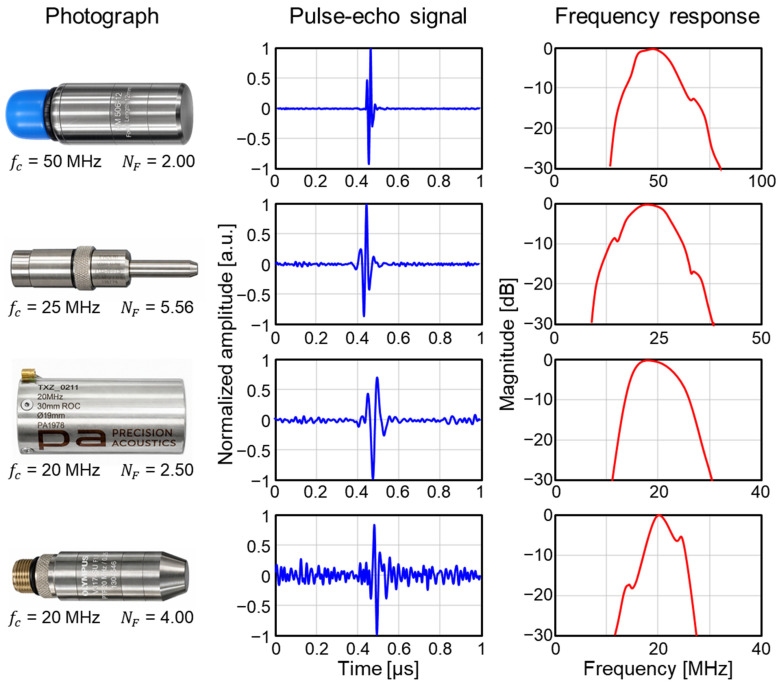
Pulse-echo response and frequency characteristics of the focused single-element ultrasound transducers used in this study. Representative A-line pulse-echo signals (blue) measured from a flat reflector are shown together with the corresponding frequency spectra (red) for the 50, 25, and 20 MHz transducers. $f_{c}$, center frequency; $N_{F}$, F-number.

A custom-designed SAM system was implemented by modifying the previously reported system [22]. The system consists of a focused single-element ultrasound transducer, a water tank for acoustic coupling, a sample holder, three linear scanning stages, a stage controller, a pulser/receiver, and a PC-based main controller (Figure 1). The system was configured to switch different single-element transducers, enabling comparative evaluation of transducers with different center frequencies and focusing conditions (Table 1). The pulse-echo signal and frequency response also confirm the characteristics of each transducer (Figure 2). To minimize the positional variation during transducer replacement, a custom holder was designed and fabricated using a 3D printer (X1 carbon, Bambu Lab, Shenzhen, China).

The system provided three-axis translation adjustment. Two motorized axes were used for automated raster scanning in the *x*–*y* directions, whereas one manual axis was used for positioning and focal alignment. The sample was immersed in deionized water, which served as the acoustic coupling medium between the transducer and the specimen. The axial position of the transducer was adjusted using a manual translational stage (XR50, Thorlabs, Newton, NJ, USA), such that the acoustic focal zone was aligned with the target imaging plane. After focal alignment, a motorized two-axis translational stage (UTM50PP1HL, Newport, Irvine, CA, USA) performed raster scanning over a predetermined imaging region. The motion of the motorized stage was controlled by a motion controller (ESP300, Newport, USA).

During scanning, the focused single-element transducer transmitted and received pulse-echo ultrasound waves. The ultrasound transmission and reception were performed using a pulser/receiver (DPR500, JSR Ultrasonics, Pittsford, NY, USA), which was operated in transmit/receive mode with a pulse repetition frequency of 200 Hz, an excitation voltage of 275 V, a pulse energy of 12.4 μJ, a damping resistance of 44 Ω, and a receiver gain of 14 dB. The same pulser/receiver settings were used for all transducers. The received signals were digitized by a high-speed digitizer (PXI-5152, National Instruments, Austin, TX, USA) installed in a PXI chassis (PXIe-1071, National Instruments, Austin, TX, USA). The received signals were sampled with 8-bit precision at a sampling rate of 500 MHz.

A custom-designed control program developed in LabVIEW 2021 (National Instruments, Austin, TX, USA) was used to manage stage scanning, trigger synchronization, and data acquisition. An arbitrary function generator (AFG1022, Tektronix, Beaverton, OR, USA) generated the trigger signals, which were shared with the pulser/receiver and the stage controller to synchronize ultrasound acquisition with stage movement. The digitized data were stored on a PC and subsequently processed for image reconstruction and further resolution analysis. The control software enabled monitoring of the acquired signals and preliminary image display during scanning.

### 2.2. Resolution Target Imaging for Quantitative Analysis

**Figure 3:**
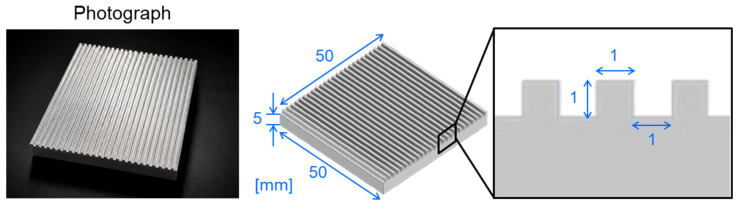
Photograph and design parameters of the resolution target consisting of alternating high- and low-height vertical stripes.

A resolution sample consisting of alternating high- and low-height vertical stripes was prepared for quantitative analysis (Figure 3). The target was fabricated by machining an aluminium plate to form periodic stripe patterns with a well-defined height difference. The spacing between adjacent vertical stripes was 1 mm, and the width of each stripe was 1 mm. Because of the height difference, the reflected pulse-echo signals from the higher region could be selectively visualized, resulting in a high-contrast SAM image resembling a binary pattern. The resulting step-like edge transitions are effective for measuring lateral resolution. To acquire images, an area of 10 × 10 mm^2^ was scanned using each transducer under identical imaging conditions. Raster scanning was performed with a lateral step size of 20 μm for both x and y directions for all transducers. Because this interval is much smaller than the theoretical lateral resolution, it ensures adequate spatial sampling of the edge profiles and consistent acquisition conditions across all transducers. The total scanning time for each image acquisition was approximately 12 min.

### 2.3. Wafer Sample Imaging for Qualitative Analysis

**Figure 4:**
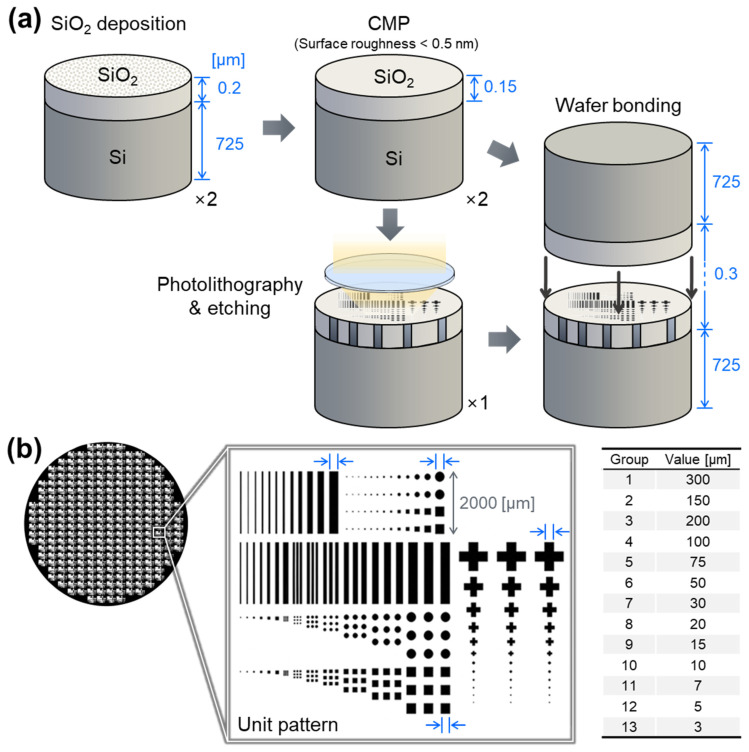
Fabrication process and geometric design of the wafer-based sample containing periodically repeated void-mimicking pattern groups. (**a**) Schematic illustration of the fabrication process. The thicknesses of the silicon substrates and SiO_2_ layers are indicated. (**b**) Layout of the 8-inch wafer sample and unit pattern group. The pattern consists of vertical-line groups, cross-shaped pads, and dot arrays. The nominal feature dimension (blue arrows) varies among the pattern groups. Si, silicon; SiO_2_, silicon dioxide; CMP, chemical mechanical polishing.

For qualitative analysis, a wafer-based sample containing void-mimicking patterns was fabricated (Figure 4a). The wafer sample was fabricated using two 8-inch silicon wafers. First, a 200 nm thick silicon dioxide (SiO_2_) insulating layer was deposited on each silicon wafer by chemical vapor deposition (CVD). Chemical mechanical polishing (CMP) was subsequently applied to reduce the surface roughness to less than 0.5 nm, resulting in a final SiO_2_ thickness of approximately 150 nm. One of the two SiO_2_-coated wafers was then patterned using a photolithography process. A photoresist mask defining the void-mimicking structures was formed on the SiO_2_ surface, followed by etching of the exposed SiO_2_ regions. This process generated recessed patterns with a nominal depth of 150 nm, including vertical-line groups, cross-shaped pads, and dot arrays with different lateral dimensions and pitches. A unit pattern group consisting of these structures was repeatedly arranged over the entire 8-inch wafer (Figure 4b). After pattern formation, the unpatterned wafer was inverted and bonded to the patterned surface, thereby enclosing the etched regions between the two SiO_2_ layers and forming internal void-mimicking cavities. These fabricated cavities were intended as controlled void-type reference targets that generate acoustic contrast through a local impedance discontinuity at the bonded interface.

Before each raster scan, the acoustic focal zone was aligned with the depth of the void-mimicking patterns. After replacement of the transducer, its initial axial position was set to the corresponding nominal focal length provided by the manufacturer. Several preliminary scans were then performed over a small region around the target depth while monitoring the pulse-echo signals and reconstructed preview images. The transducer position was iteratively adjusted using the manual z-axis stage until the echo amplitude from the target layer was maximized and the pattern boundaries showed the greatest sharpness. A single unit pattern group was then imaged using raster scanning. For each transducer, an area of 20 × 20 mm^2^ was scanned with a lateral step size of 0.02 mm in both the x and y directions. The total scanning time for each acquisition was approximately 48 min.

### 2.4. Signal and Image Processing

All the signal and image processing was performed using a unified pipeline implemented in MATLAB R2024a (MathWorks, Natick, MA, USA). The raw signals acquired at each scanning position were first band-pass filtered to suppress out-of-band noise. The filter ranges were set to 25–75 MHz, 10–40 MHz, and 10–30 MHz for the 50 MHz, 25 MHz, and 20 MHz transducers, respectively. Envelope detection was then performed using the Hilbert transform to obtain the amplitude profile of the signal.

The sample surface was automatically detected from the maximum envelope amplitude within the first 1000 samples of each A-line. The resulting surface-position map was spatially smoothed using a 20 × 20 median filter. Each A-line was then axially aligned using the surface-position map, thereby flattening the sample surface before image reconstruction. To improve the axial sampling density of the envelope signal, piecewise cubic Hermite interpolating polynomial (PCHIP) interpolation was applied.

After interpolation, an axial gate with a width of 20 μm was selected around the target depth to isolate the reflected signals from the region of interest. A maximum amplitude projection (MAP) was then performed along the axial direction within the selected gate, generating a two-dimensional image for each scan. For visualization and comparison, the reconstructed MAP images were further interpolated in both *x* and *y* directions using spline interpolation to achieve improved pixel resolution. For image display, each MAP image was independently normalized to the range of 0–1 using linear min–max normalization and displayed using the same normalized scale. Therefore, the displayed grayscale represents relative signal intensity within each image rather than absolute amplitude across transducers. The same processing was applied to all data to ensure consistent comparison among transducers.

## 3. Results and Discussion

### 3.1. Quantitative Resolution Assessment

**Figure 5:**
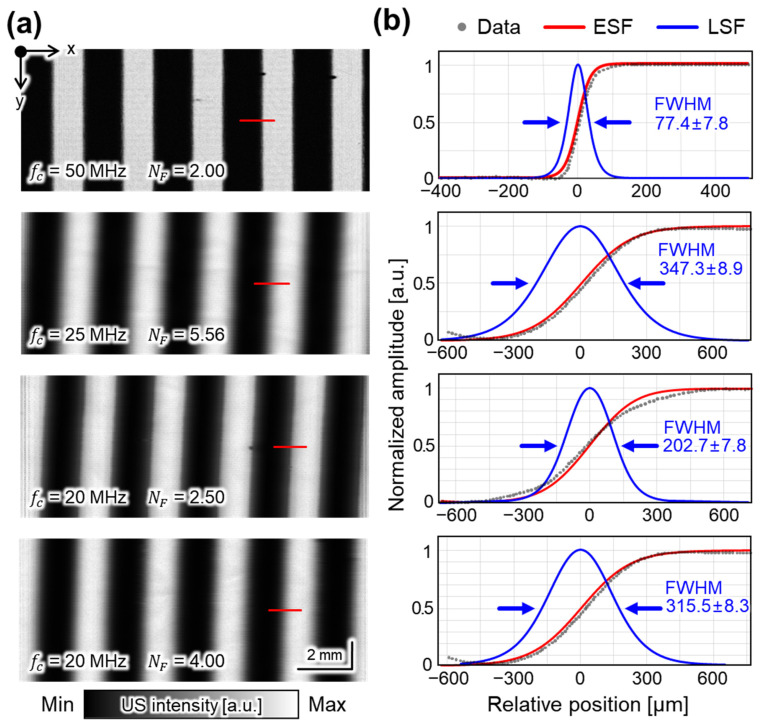
Quantitative lateral resolution analysis using a striped resolution target. (**a**) Reconstructed SAM images of the striped resolution target using focused single-element transducers with different center frequencies and F-numbers. (**b**) Representative ESF and LSF profiles extracted from the stripe edges (red lines in (**a**)) for lateral resolution analysis. The FWHM of the LSF was used to quantify the lateral resolution of each transducer. SAM, scanning acoustic microscopy; US, ultrasound; $f_{c}$, center frequency; $N_{F}$, F-number; ESF, edge spread function; LSF, line spread function; FWHM, full width at half maximum.

To compare the imaging performance of transducers, the striped resolution target was imaged using the four focused transducers (Figure 5a). The image acquired with the 50 MHz transducer showed clearly resolved vertical stripes, with sharp edge transitions and high contrast between the high- and low-height regions. In contrast, the image acquired with the 25 MHz transducer showed markedly blurred edges, with substantial intensity spreading between adjacent stripes. Interestingly, the 20 MHz transducer provided intermediate image quality despite having the lowest center frequency among the four transducers. This result suggests that the lateral resolution of SAM is not only determined by the center frequency alone, but also strongly affected by the focusing condition, represented by the F-number.

For quantified comparison, the lateral resolution of each image was measured by calculating the full width at half maximum (FWHM) of the line spread function (LSF). At each stripe edge, the intensity profile was normalized to the range of 0–1 after subtraction of its minimum value. PCHIP interpolation and Boltzmann fitting were then applied to obtain the edge spread function (ESF). The corresponding LSF was derived by differentiating the fitted ESF and was subsequently fitted with a Gaussian function (Figure 5b). The FWHM of the Gaussian-fitted LSF was extracted as the lateral resolution. To improve statistical reliability, this procedure was repeated at 100 independent positions along the stripe edges. The mean and standard deviation of the FWHM values were calculated to represent the lateral resolution of each transducer.

The 50 MHz transducer produced the narrowest ESF transition and consequently the smallest LSF width, resulting in the best lateral resolution of 77.4 ± 7.8 μm. In contrast, the 25 MHz transducer yielded the broadest LSF and the poorest lateral resolution of 347.3 ± 8.9 μm. Among the two 20 MHz transducers, the transducer with an F-number ($N_{F}$) of 2.50 achieved a lateral resolution of 202.7 ± 7.8 μm, whereas the transducer with a larger F-number of 4.00 showed a broader LSF and a lateral resolution of 315.5 ± 8.3 μm. These measured values were consistent with the corresponding theoretical lateral resolutions in Table 1.

The influence of center frequency can be assessed by comparing the 50 MHz (2.00 $N_{F}$) and 20 MHz (2.50 $N_{F}$) transducers, which had relatively similar F-numbers. The 50 MHz transducer provided better lateral resolution, consistent with its shorter acoustic wavelength. Although the F-numbers were not identical, this comparison supports the expected improvement in lateral resolution with increasing frequency under comparable focusing conditions.

The comparison between the two 20 MHz transducers provides a more direct assessment of the influence of F-number at the same center frequency. Although both transducers had the same nominal acoustic wavelength, the transducer with an F-number of 4.00 exhibited poorer lateral resolution than that with an F-number of 2.50. This result indicates that increasing the F-number broadens the focal beam and degrades lateral resolution. An overall trend of progressively poorer lateral resolution was also observed for increasing F-number: 20 MHz (2.50 $N_{F}$), 20 MHz (4.00 $N_{F}$), and 25 MHz (5.56 $N_{F}$). Notably, the 25 MHz transducer showed poorer resolution than both 20 MHz transducers despite its higher center frequency. According to the diffraction-limited approximation, lateral resolution is proportional to both acoustic wavelength and F-number. Therefore, the benefit of the shorter wavelength at 25 MHz was offset by its substantially larger F-number. These comparisons demonstrate that center frequency alone is insufficient for predicting SAM resolution and that focusing geometry must be considered together with operating frequency.

### 3.2. Qualitative Image Analysis

**Figure 6:**
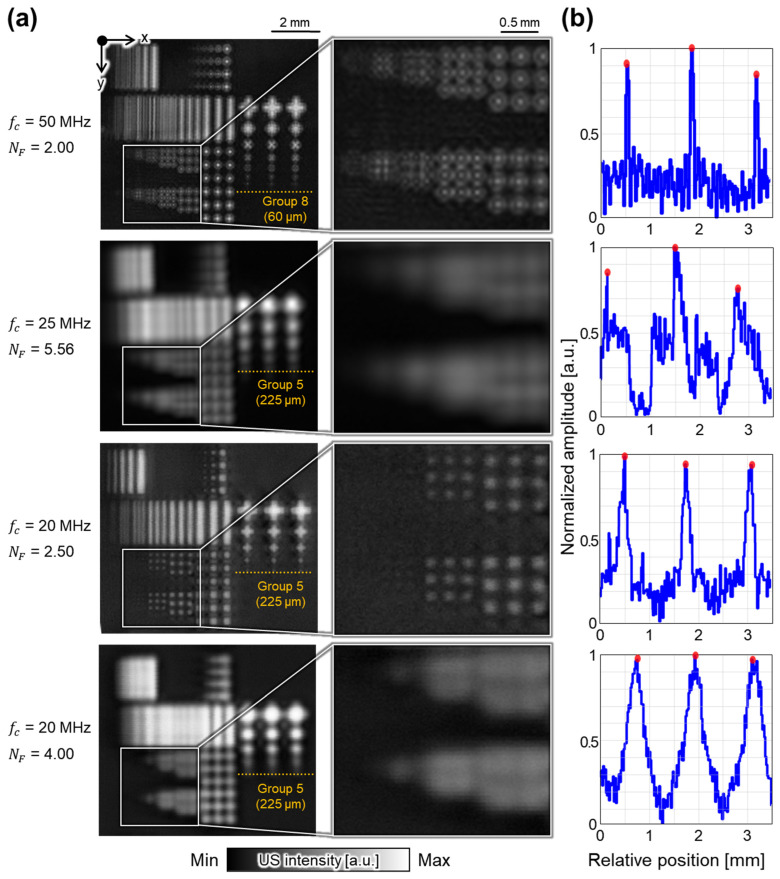
Qualitative evaluation of SAM imaging performance using a wafer-based sample with void-mimicking patterns. (**a**) Reconstructed SAM images acquired using focused single-element transducers with different center frequencies and F-numbers. Enlarged images of the selected regions show detailed structures in the white boxed regions. (**b**) Line profiles extracted along the yellow lines of the cross-shaped pads for Groups 8 or 5. Red circles represent peak signals detected from cross-shaped features. SAM, scanning acoustic microscopy; US, ultrasound; $f_{c}$, center frequency; $N_{F}$, F-number.

Based on the quantitative lateral resolution, the imaging performance of the transducers was further evaluated using a wafer-based sample, which contained void-mimicking patterns designed to emulate internal defects that may occur in semiconductor devices. The pattern layout included vertical-line groups of varying pitches, cross-shaped pads, and dot arrays with different feature sizes. SAM images acquired using the four transducers successfully visualized the overall arrangement of the void-mimicking patterns (Figure 6a). However, the image quality and resolvable feature size differed depending on the transducers.

The SAM image acquired with the 50 MHz transducer most closely reproduced the reference layout of the wafer sample. The void-mimicking patterns were clearly resolved with sharp boundaries and high contrast between adjacent features. In particular, the cross-shaped pads and dot arrays retained their geometries, indicating superior lateral resolution. Among the cross-shaped pads, the smallest visible group was Group 8 (Figure 6b), which has an ideal feature size of 60 μm. Considering fabrication tolerance, this result is qualitatively consistent with the lateral resolution of 77.4 ± 7.8 μm, obtained from the striped resolution target in the previous section.

The two 20 MHz transducers provided a direct qualitative comparison of the effect of F-number at the same center frequency. The transducer with an F-number of 2.50 preserved the geometries of the void-mimicking patterns more clearly than the transducer with an F-number of 4.00. Although both transducers had the same nominal acoustic wavelength, the image acquired with the larger F-number showed broader features, reduced boundary sharpness, and poorer separation between closely spaced patterns. This observation agrees with the quantitative resolution measurements and further indicates that an increase in F-number degrades lateral image quality even with the same operating frequency range.

The image acquired with the 25 MHz transducer showed pronounced blurring, particularly in the smaller patterns. Although the overall pattern arrangement could still be recognized, fine features were poorly distinguished, and the cross-shaped pads lost their characteristic geometry. Notably, the 25 MHz transducer provided poorer image quality than both 20 MHz transducers despite its higher center frequency. The benefit associated with its shorter acoustic wavelength was offset by its substantially larger F-number, resulting in a broader acoustic focus and reduced lateral resolving capability.

To further compare the qualitative imaging performance, line profiles were extracted across cross-shaped pads with a nominal feature size of 225 μm (Figure 6b). Peak signals corresponding to the cross-shaped structures were detectable, but the profiles became progressively broader and exhibited less distinct peak separation as the F-number increased. The 50 MHz (2.00 $N_{F}$) transducer showed the clearest separation among the transducers. The 20 MHz (2.50 $N_{F}$) transducer still showed a clear separation, whereas the 20 MHz (4.00 $N_{F}$) transducer exhibited broader peaks. The 25 MHz (5.56 $N_{F}$) transducer showed the poorest separation and stronger side-lobe-like intensity fluctuations, which may reduce the distinguishability of adjacent features when the spacing between structures is small. These profile trends were consistent with the quantitative lateral-resolution measurements obtained using the striped resolution target.

Overall, the wafer-based imaging results confirm that the trends observed in the resolution target experiment are also evident in semiconductor-like specimens. The 50 MHz transducer with the smallest F-number provided the best image quality, whereas the 25 MHz transducer with the largest F-number produced the most degraded images. The two 20 MHz transducers demonstrated that image quality degraded as the F-number increased at the same center frequency. These findings further demonstrate that center frequency alone is insufficient for predicting practical SAM image quality and that both acoustic wavelength and focusing geometry should be considered when selecting transducers for target-specific semiconductor inspection.

## 4. Conclusions

In this study, the way in which center frequency and F-number jointly affect the lateral resolution of SAM was investigated using a custom-designed system. Four focused single-element transducers were compared using a striped resolution target and a wafer-based sample containing void-mimicking patterns. The measured lateral resolutions were consistent with the theoretical trend based on the acoustic wavelength and F-number. In particular, the comparison between the two 20 MHz transducers showed that a larger F-number substantially degraded lateral resolution at the same center frequency, indicating that the F-number affects the lateral resolution. The wafer-based imaging results showed the same qualitative tendency. Overall, a higher center frequency did not necessarily result in better lateral resolution when the F-number was relatively large. These results indicate that transducer selection for SAM-based inspection should consider both parameters together.

However, this study has several limitations. The comparison was empirically performed using only four available transducers, and frequency and F-number were not independently controlled across all configurations. Other transducer parameters, including focal length, aperture size, and frequency bandwidth, also differed among the transducers. Therefore, the present study should be interpreted as a representative experimental comparison rather than a fully systematic transducer optimization study or a general guideline for SAM transducer selection. In addition, the transducer used in this study operated below the frequency range commonly used for high-resolution semiconductor inspection, where transducers above 100 MHz are often required. Finally, the wafer-based sample contained defect-mimicking patterns rather than process-induced defects in actual semiconductor devices.

Despite these limitations, the present work provides a practical experimental basis for understanding transducer-dependent image quality in SAM-based semiconductor inspection. The results show that the F-number can substantially alter the resolution trend expected from the center frequency alone. The consistent trends obtained from both quantitative resolution assessment and qualitative imaging provide useful reference data for evaluating representative transducer configurations. Accordingly, the present findings should be regarded as a basis for future systematic investigations rather than as a complete framework for transducer selection.

Therefore, future work should include a broader and more systematically selected set of transducers, including higher-frequency transducers suitable for semiconductor inspection. Independent control of frequency, F-number, focal depth, and bandwidth will be important for establishing practical transducer selection guidelines. In addition, dedicated multilayer samples containing identical resolution features at multiple controlled depths will be required to characterize depth-dependent changes in lateral resolution, penetration capability, and imaging performance throughout the focal zone. Further studies using actual semiconductor devices and packaged structures containing naturally occurring or process-induced buried voids, delamination, and micro-cracks will also be necessary to validate the proposed evaluation approach under realistic inspection conditions. In addition, improved transducer alignment, image registration, high-speed scanning, and automated image analysis may further enhance the practical applicability of SAM for semiconductor manufacturing inspection.

## Figures and Tables

**Table 1:** Characteristics of the focused single-element ultrasound transducers used in this study. The acoustic wavelength is calculated from the center frequency using an assumed sound speed of 1500 m/s for consistency in theoretical comparison. $f_{c}$, center frequency; $l_{f}$, focal length; D, aperture diameter; $N_{F}$, F-number; λ, acoustic wavelength; $\delta_{L}^{*}$, theoretical lateral resolution.

Model	Manufacturer	$f_{c}$ [MHz]	$l_{f}$ [mm]	D [mm]	$N_{F}$ [a.u.]	λ [μm]	$\delta_{L}^{*}$ [μm]
HM506-12	OHLABS, Busan, Republic of Korea	50	12	6	2.00	30.0	61.2
V324-N-SU	Evident Scientific, Tokyo, Japan	25	35.33	6.35	5.56	60.0	340.3
TXZ_0211	Precision Acoustics, Dorchester, UK	20	30	12	2.50	75.0	191.3
V317-SU	Evident Scientific, Japan	20	25.4	6.35	4.00	75.0	306.0

## Data Availability

The data presented in this study are available on request to the corresponding author.
